# Involvement of adolescent representatives and coresearchers in mental health research: Experiences from a research project

**DOI:** 10.1111/hex.13383

**Published:** 2021-11-10

**Authors:** Petter Viksveen, Nicole E. Cardenas, Maya Ibenfeldt, Laia G. Meldahl, Lou Krijger, Julia R. Game, Maren McLean Andvik, Oliver Cuddeford, Samuel Duerto, Murad Mustafa, Mathias Tong

**Affiliations:** ^1^ Department for Quality and Health Technology, Faculty of Health Sciences, SHARE—Center for Resilience in Healthcare University of Stavanger Stavanger Norway; ^2^ School of Psychology (Psychology) University of Aberdeen Aberdeen Scotland; ^3^ Faculty of Biology, Medicine and Health (Pharmacology) University of Manchester Manchester UK; ^4^ Faculty of Health Sciences (Psychology) UiT Arctic University of Norway Tromsø Norway; ^5^ ESCP Business school, Business Management Ecole Supérieure de Commerce de Paris Paris France; ^6^ Faculty of Medicine (Medicine), Institute of Medicine Pomeranian Medical University Szczecin Poland; ^7^ College of Medicine, Veterinary and Life Sciences (Zoology) University of Glasgow Glasgow Scotland; ^8^ School of Arts and Humanities, Faculty of Art Design and Architecture (Architecture) University of Huddersfield Huddersfield UK; ^9^ Faculty of Philosophy, Theology and Religious Studies (Philosophy, Politics and Societies) Radboud Univeirsty Nijmegen The Netherlands; ^10^ Faculty of Health Sciences (Nursing) University of Stavanger Stavanger Norway; ^11^ Department of Chemical Engineering and Analytical Science (Chemical Engineering) University of Manchester Manchester UK

**Keywords:** adolescents, involvement, mental health, shared decision‐making, youth

## Abstract

**Introduction:**

In spite of adolescents' rights to be involved in decisions that concern their health and life, limited research has been published reporting on their involvement in mental health research. Therefore, we aim to present experiences and reflections based on the involvement of adolescents in mental health research, to describe the collaborative relationship between researchers and coresearchers, including the values that underpin their collaboration.

**Methods:**

An autoethnographic approach was used, combined with group reflections. The process was jointly developed, carried out and analysed by adolescent coresearchers and the project's lead researcher over a period of 2 years. The article is jointly authored by the researcher and the ten coresearchers.

**Results:**

Six themes were developed to describe our collaborative relationship, resulting in the involvement of adolescents in decisions about research priorities; in planning and carrying out the research; through to analysis, dissemination and communication of results. The themes include: (1) Commitment motivated by altruism, personal interests and a common purpose; (2) Inclusiveness and support to reduce social uncertainty and strengthen collaboration; (3) Reduced power differentials while ensuring clarity of roles and tasks; (4) Diversity in representation to expand the perspectives of ‘the adolescent voice’; (5) Self‐determination—supporting adolescents' involvement in decision‐making processes; and (6) Flexible and systematic project management. The themes describe the collaboration, the underlying values and motives, the challenges faced and how they were overcome.

**Conclusion:**

This self‐reflective process describing a 4‐year collaborative research project resulted in the development of recommendations for involving adolescents in mental health research. The recommendations could potentially contribute to a change of ‘research culture’ to expand the currently limited involvement of adolescents in research.

**Patient or Public Contribution:**

Adolescents have contributed as coresearchers through all phases of the research project and as coauthors of this article, including planning, participation in the self‐ and group‐reflective processes, analysis and authoring the article.

## 1 INTRODUCTION

According to the United Nations' Convention on the Rights of the Child, adolescents should be able to enjoy the highest attainable standard of health (Article 24).^1^ Their rights should be respected irrespective of their and their parents' or legal guardians' ‘*race, colour, sex, language, religion, political or other opinion, national, ethnic or social origin, property, disability, birth or other status*’ (Article 2). Furthermore, one of the four general principles of the UN Convention on the Rights of the Child (Article 12) states that children should have the right to express their views and their opinion should be given due weight. The Council of Europe points out that each country should ensure that adolescents are listened to and involved in any decisions affecting them.^2^ In its *Manual on the Revised European Charter on the Participation of Young People in Local and Regional Life*, the Council of Europe describes different levels at which youth can be involved, ranging from ‘nonparticipation’ through to being informed, consulted, or involved in decision‐making processes and sharing decision‐making power.^3^

Adolescents may be involved and influence healthcare services at an individual, systems or political level. At the individual level, they may influence their own healthcare, for example, by stating their wishes for treatment and support, or by being involved in decision‐making processes to improve the services they receive. At the systems level, they can influence the healthcare services for other adolescents, for example, through the development, review or improvement of hospital psychiatric departments. They may at the political level influence decisions made by regional or national healthcare authorities. The results of our systematic review on user involvement in adolescents' mental healthcare suggest that there is some research evidence reporting on adolescents' involvement at the individual level, where they are heard and take part in decision‐making processes, whereas we found less evidence reporting on adolescents' involvement at the systems level.^4^ Involvement in research focusing on adolescent mental health, such as studies assessing existing healthcare services, contributing to the improvement of existing services or development of new interventions, may be considered involvement at the systems level. Alternatively, it could be considered a fourth level. Others have stressed the importance of involving youth in order for their perspectives to be integrated into research projects. For example, McDonagh and Bateman^5^ suggested that children and adolescents could be involved at various stages of research processes, such as contributing to setting the research agenda, developing research projects, being involved as participants or coresearchers and disseminating results. However, there is a paucity of research literature reporting on the involvement of youth in mental health research.^6^, ^7^ Others found that reasons for not involving youth in research may be due to lack of resources, researchers' concerns about adolescents' well‐being, feeling loss of control of the research process or that peer researchers might not value the inclusion of nonresearchers.^8^

Although examples of meaningful involvement of adolescents in research do exist, there is still limited research reporting on the collaborative relationship between researchers and adolescent coresearchers in mental health research. Furthermore, research focusing on values in adolescent mental health research is lacking. The aim of this article is therefore to present experiences and reflections based on the involvement of adolescents in mental health research, with a particular focus on describing the collaborative relationship between the lead researcher and coresearchers, including the values underpinning the collaboration. We will also present our recommendations for involving adolescents in mental health research.

### 1.1 The project background and context

The research project, entitled *InvolveMENT*, was established in 2017 and aims to contribute to improving mental health services for adolescents. Participation of adolescents throughout all phases of the research has been a key priority since the start of the project (described later).

The research project is based within *SHARE—Centre for Resilience in Healthcare* at the *Faculty of Health Sciences*, *University of Stavanger*, in Norway. The research centre was formally established in March 2017 and was based on a patient safety research programme running from 2011 to 2017. It aims to be an internationally recognized research centre in the field of quality and safety of healthcare systems using a new and comprehensive resilience in healthcare (RiH) framework. It includes over 70 researchers in various clinical fields and at different service levels, including primary, secondary, prehospital and transitional care. Patient and stakeholder involvement is one of the centre's six strategic research priorities (2018–2022).^9^ Patients, carers, citizens and other major stakeholders are included as equal partners in the centre's research activities, and a stakeholder reference group provides recommendations to the research centre. One of the adolescent mental health research project coresearchers (L. G. M.) also became a member of the research centre's stakeholder panel.

Adolescent representatives and coresearchers were recruited to the research project from upper secondary school and mental health services. They were not participants in the study, but were first included as representatives and later changed status to coresearchers. Their participation was therefore regulated by legislation relating to their general rights for self‐determination, rather than legislation relating to medical and health research. The individual's right to self‐determination is a central principle in Norwegian legislation. Any competent person has such rights from the age of 18. Moreover, the rights for self‐determination gradually increases with age and from the age of 15, adolescents have the independent right to make decisions concerning their education and work life.^10^, ^11^ All adolescents were at least 15 years and could therefore make the decision to join the project without seeking their parents' or legal guardians' consent.

The roles and responsibilities of the University, the researchers and adolescent representatives were agreed upon at the onset of the collaboration (Appendix SA). This included issues, such as adolescents' ownership of their own academic material and their right to leave the project at their own discretion at any given point in time should they wish to do so; the researchers' right to make final decisions concerning the research and their ownership of academic material produced as part of the research project; as well as the University's financial responsibilities. It also addressed issues such as remuneration and confidentiality. Although the agreement aimed to clarify the rights and responsibilities of all parties, it also expressed the intention to develop a collaborative relationship, for example, by pointing out that adolescents would be consulted to integrate their views and opinions into the research. As representatives, they were asked for and provided their comments to research planned and carried out by researchers. Their status was changed to coresearchers when they initiated, planned and carried out parts of the research project with the support of the project researchers. This transitional process is further described in Section Results, 3.

The lead researcher (P. V.) is an Associate Professor in Healthcare Sciences. He has continuously collaborated with adolescent representatives and coresearchers through all phases of the research. The project receives financial support for some of the activities through the *Resilience in Healthcare* project funded through the *Research Council Norway* (FRIPRO TOPPFORSK funding stream, grant agreement no. 275367).

### 1.2 Adolescents' contributions to the research

Adolescents participated in setting research priorities, planning research (systematic review, qualitative study, surveys and trials), developing survey questionnaires, recruiting participants, analysing results, dissemination (academic) and communication (nonacademic), and by initiating some of the research. Involvement varied for different parts of the research project and took place at three levels, as suggested by Oliver et al.^12^: consultation, collaboration and control. Consultation meant that adolescents' opinions were sought by researchers; collaboration implied a closer interactive process where decisions were jointly agreed; whereas adolescents initiated and made their own decisions when exerting control, but with the input and support of the researcher. An overview of adolescents' most important contributions to the research project is presented in Table 1.

**Table 1:** Areas of adolescents' contribution to the research project

Research area	What adolescents contributed to	Who contributed?
Research priorities	Setting the research agenda:	Adolescent representatives and coresearchers, 150 second‐year secondary school students, 400 seminar participants
	To assess factors that affect adolescents' choice to seek help for their mental health To assess healthcare services and barriers to adolescents' help‐seeking behaviours To develop means to improve access to and quality of mental health services, including for minority groups To improve adolescents' involvement in their own healthcare To integrate leisure time activities to strengthen social networks and improve adolescents' mental health	
Funding applications and policies	Five separate funding applications were submitted to two different funding bodies, as well as adding leisure time activities to a 4‐year research project. The project will further develop and test this and an eHealth intervention in a population cohort survey, a qualitative study with ethnic minority groups and a randomized controlled trial to test the acceptability, effectiveness and cost‐effectiveness of interventions. The review panel stated they: ‘*[…] were pleased that young people in particular appear to be involved in a meaningful way in all phases of this proposal*’.	Representatives and coresearchers
	Contributed to the research centre's *Patient and Stakeholder Involvement (PSI) Strategy (2020–2022)* ^13^	
Mental health surveys	Adapted content and layout of questionnaires used in four cross‐sectional surveys (*n* = 2500) based on validated outcome measures, to strengthen the relevance to adolescents' needs	Coresearchers
	Initiated and carried out two surveys with secondary school students (*n* = 159)	
	Recruitment of participants in three surveys (*n* = 1600)	
Systematic review	Planning and carrying out a systematic review, with decisions on article inclusion, development of a thematic synthesis to present adolescents' involvement in mental healthcare, and writing protocol and results articles^4^, ^14^	Coresearchers
Mental health seminar	Initiated and co‐organized a 1‐day seminar (400 participants) in September 2019 focusing on the stigma associated with mental health and mental health service use, at the university, funded through the research centre's *Resilience in Healthcare* project and the *World Mental Health Day*. Adolescents gave six of seven presentations	Representatives and coresearchers
	Collected data from participants to gather their perspectives to develop recommendations for the prevention of mental health stigma	
Dissemination and communication	Coauthors of five scientific articles (two published, three submitted)	Representatives and coresearchers
	Presentations at six seminars and conferences:	
	*5th Nordic Conference on Research in Patient Safety and Quality* in Healthcare, Copenhagen 2018 (oral presentation) *5th International Conference on Youth Mental Health*, Brisbane, Australia 2019 (coauthors) Seminar on user involvement for children and youth, *Regional Centre for Child and Youth Mental Health and Child Welfare*, *UiT The Arctic University of Norway* 2020 (oral presentation) Participative research seminar, *University of Geneva*, Switzerland 2020 (oral presentation) *LEADS Conference for Learning Enhancement and Academics Development Service*, *University of Glasgow*, UK 2021 (oral presentation) *Youth Data population survey kick‐off seminar*, *Korus Vest Stavanger*, Norway 2021 (oral presentation) Media interviews: newspaper, radio and TV in 2019 Discussion with the *Minister of Health* and the *Minister of Children and Equality* who were visiting the university in 2018	

## 2 METHODS

Ten adolescent coresearchers and the project's lead researcher are the authors of this article. We used an autoethnographic approach,^15^ combined with paired and group‐reflective processes to deepen our understanding of our collaborative relationship. The autoethnographic method involves the active participation of researchers through a self‐reflective process that may evoke memories, thoughts and feelings. Therefore, it differs from an autobiography, which involves recollection of past experiences; and is more like an ethnographic approach studying aspects, such as values, beliefs and experiences within a culture, with the distinction that the individuals studied are the researchers themselves.^16^ By using an autoethnographic approach, we could go beyond merely ‘telling our story’ and engage in a deeper reflective process to explore our own experiences, values and beliefs. Hence, we served as the primary tool in both data collection and analysis.

The research process was initiated by the lead researcher (P. V.) and the two adolescent representatives (J. R. G. and N. E. C.) who first joined the project in 2017. The remaining eight adolescents (L. G. M., L. K. P., M. I., M. M., M. M. A., M. T., O. C. and S. D.) were involved after they joined the project in 2018 and 2019. In the period from August 2018 to July 2020, we used a combination of several individual reflections, paired reflective processes and group discussions to explore our experiences and views, and to develop our recommendations for involving adolescents in mental health research. The self‐reflective processes were based on questions raised about the research project and the nature of the collaboration within the research group. Examples of questions included: ‘*What were your motives and values for joining this project?*’ and ‘*How can we effectively involve teenagers as representatives or co‐researchers in a mental health research project?*’ A complete list of questions may be found in Appendix SB. All were encouraged to describe in their own words and provide examples from the research project to illustrate experiences, thoughts and opinions. Paired reflective processes and group discussions were used to raise additional thoughts.

Notes were kept on both self‐reflective and paired processes, and arising issues were brought up in group discussions. We commented and reflected in pairs on each other's notes. This could for example involve our thoughts on underlying values and motives for participating in the project or our reflections on our interaction with each other. Thoughts on what we considered key issues were discussed during meetings, and notes of meetings were kept. We learned from each other and gained a deeper understanding of our thoughts and values. The content of each discussion area was gradually developed. Descriptions of the content of each theme eventually resulted in proposals for themes, which were agreed through consensus processes during group meetings. We used our narratives to develop themes describing our experiences, as well as the nature of our collaborative relationship. We subsequently developed our recommendations for involving adolescents in mental health research.

## 3 RESULTS

Six themes describe our *collaborative relationship*, including the *underlying values and motives for participating in the project, the challenges faced* and *how they were overcome*. The themes that have been developed through our self‐reflective processes, paired and group discussions are:

1. Commitment motivated by altruism, personal interests and a common purpose.
2. Inclusiveness and support to reduce social uncertainty and strengthen collaboration.
3. Reduced power differentials while ensuring clarity of roles and tasks.
4. Diversity in representation to expand the perspectives of ‘the adolescent voice’.
5. Self‐determination ‐ supporting adolescents' involvement in decision‐making processes.
6. Flexible and systematic project management.

We use examples to illustrate how each of the six themes has been essential to our collaborative relationship. Although we have agreed on and present the results in the following sections as a collective group, we will clarify when we refer to perspectives presented by one or several coresearchers, or the lead researcher.

### 3.1 Commitment motivated by altruism, personal interests and a common purpose

An important value held by us all was a selfless interest in contributing to the welfare of adolescents. Were the sources of such an altruistic mindset due to biological factors or sociocultural conditioning? How did our own past experiences influence our motives? Or were the motives not so altruistic and more fuelled by a form of self‐gratification by contributing to (hopefully) improve the mental health services for adolescents?

We agreed that there was an element of ‘altruism’ and we considered empathy to be an important quality. When asked the question: ‘*What value is needed to participate in the project?*’ one coresearcher emphasized: ‘*[…] empathy for others. I wouldn't be able to do this work if I didn't have a drive to help others*’. Another coresearcher answered: ‘*If you've been involved negatively with healthcare services, you feel empathy for others who experience the same. If you've had a positive experience […] you might think: I've had opportunity – what can I do to help them give the opportunity?*’

We believed that our past experiences might be helpful to the project and that we were in a position to understand what an adolescent may undergo when trying to access and receive professional help. Through the research project, we were united by wanting to encourage adolescents with mental health problems to consider seeking help and support. Moreover, we wished to improve mental healthcare services, so more adolescents could view them as a positive contribution to their mental wellness. The fruits of our labour were realized during our seminar in 2019 where one member reflected: ‘*There was a moment where it all became real to me, it was after our mental health stigma seminar for adolescents, and we were looking through the participants' feedback and someone had commented: You made me realise I should seek help*’. This was our main motivation when organizing the seminar. We aimed at sharing our varying experiences with the stigma associated with mental health, with topics ranging from mental health associated with coming out of the closet to arriving in the country as a refugee, in hope that others would feel less burdened by stigma and seek appropriate help.

However, we also realize we might give the impression of a too ‘glorified’ picture of our altruistic motivations. Our different backgrounds and experiences contributed to each one of us having personal reasons for our participation in the research. Participation in the project supported our own personal interests and it had certain benefits. It could increase our awareness, for example, about mental health and research; and expand our knowledge and understanding to better handle mental health issues. Another advantage could be to develop skillsets, such as communication and collaboration skills, carrying out surveys, organizing seminars and media and presentation skills. Moreover, it could expand our social network; and remuneration for participating could be an incentive.

Some of these benefits have also been highlighted by others.^5^ Through our participation in the research project, some of us fulfilled an obligation to participate in extracurricular activity required as part of our secondary school degree. Moreover, participation in the project was beneficial to some of us who applied to be accepted as university students abroad, two of us were invited to participate in a reference group in another research project, and one member got a position as a research assistant at another university.

### 3.2 Inclusiveness and support to reduce social uncertainty and strengthen collaboration

It was initially a daunting experience for us as teenagers to become involved in a University research project. First, we felt unprepared. Although most of us have experiences with the use of mental health services, we feared we had insufficient knowledge about the inner workings of the services and how to conduct research in this field. ‘*Having had minimal experience in this subject prior to joining, one challenge was having the confidence to speak, especially in the presence of people more knowledgeable than me*’. This caused concerns about our views and opinions being de‐emphasized or dismissed. Second, not knowing the researcher and knowing only some or none of the other adolescents added to the feeling of uncertainty about what to expect and it caused some initial reluctance to openly discuss mental health issues within the group. Moreover, adolescents joined the project at different points in time. Some felt uncertain about their role as they were new to the project and others had already been involved for a year or more. Third, there is a mix of Norwegian and non‐Norwegian, bi‐ and multicultural backgrounds, and a variety of mother‐tongues, including Arabic, Dutch, English, French, Kurdish, Norwegian and Spanish. These differences contributed to feelings of uncertainty, hesitation to contribute to group discussions and a sense of isolation for some of us. Although all of us could speak and understand both Norwegian and English to some extent, the degree to which we felt comfortable with speaking both languages varied, particularly as our discussions focused on topics that required more than ‘everyday language skills’. For these reasons it was somewhat challenging, at least initially, to communicate about issues that could be of a personal nature, when discussing the use of mental health services and research.

In spite of our initial uncertainties and concerns, our experiences with the collaboration were positive. We soon felt quite free to openly express our opinions, with each other and with the lead researcher, even when discussing issues in the field of mental healthcare that were somewhat sensitive or potentially controversial. This was facilitated by the inclusiveness in the project, where the lead researcher was genuinely interested in our opinions and over time we saw that they were also used to inform decision‐making processes. For example, our suggestions to carry out mental health surveys with our peer students, to organize a seminar by adolescents for adolescents on mental health stigma and to include social support networks as an intervention in a research funding application, were all implemented. Inclusiveness within the group was also illustrated by our implementation of a ‘going around the table’ routine so each one of us could express their opinions one at the time, before we started a more open, nondirected discussion; and allowing members to speak either in English or Norwegian. Moreover, our intent to be inclusive was further displayed by participation with the lead researcher at secondary school meetings where we shared our experiences and encouraged more adolescents to join the project.

As lead researcher of the project and given the fact that I had no prior experience with involving adolescents in research, I was uncertain whether they would be interested in participating, and how and to what extent they would be willing and able to do so. I encouraged all to contribute with their views and opinions through our discussions, listened attentively to them and included them in decision‐making processes. A scaffolding approach was used that initially involved information provision (e.g., about research methods in general and the research project in particular) and training (e.g., a workshop about systematic reviews) to help them to increase their knowledge about mental health and research. As they became more familiar with the project and each other, and as their competence levels increased, I took a more facilitating role where discussions would ‘flow more freely’. Over time, adolescents were given more freedom to carry out projects they felt passionate about, such as organizing the mental health seminar. Increasing their decision‐making power allowed them to form closer bonds with each other, as well as giving me, as the lead researcher, an opportunity to see what they were capable of. One adolescent mentioned that ‘*being given the opportunity to plan the seminar on our own, we felt our opinions really were valued, and we were partners in a project, not just fulfilling a quota*.’

### 3.3 Reduced power differentials while ensuring clarity of roles and tasks

When first joining the project as representatives, we had an expectation that the researcher–adolescent relationship would resemble a teacher–student or boss–employee relationship where the researcher would be ‘superior’ in some ways. Being undermined by authority figures was a common experience for us. We expected to be met with misconceptions of lacking experience and knowledge. We did not expect a two‐sided collaborative relationship where our thoughts and perspectives were emphasized in research decisions. ‘*I assumed we would sit in a room once a month and talk about our ideas while a researcher took notes, but I did not expect to be as involved as we have been*’. One of the first roles we took on was as coauthors of a systematic review assessing user involvement in adolescents' mental healthcare. As part of this process, we were consulted on articles where the other coauthors—who were all university researchers—had differing opinions. We were not used in everyday life to professionals actually listening to what we had to say. Our views were taken into consideration and consensus decisions were eventually reached. We were also surprised when we were trusted to organize and run a seminar. This helped us understand our importance in the roles of adolescent representatives and coresearchers.

These experiences add to what we already described: A sense of inclusion in the project and active involvement in decision‐making processes. One coresearcher stated: ‘*This project has given me hope that researchers aren't as portrayed in the sense that they overlook the voice of the teenager, and that someone is […] trying to find a compromise between realistic advancements but based on what teenagers say they want themselves*’. The absence of a stricter hierarchical structure ‘*[…] has made communication with each other easy and has facilitated the good teamwork*’. It contributed to reducing our initial perception of power differentials. However, some coresearchers questioned whether this also led to prolonged and, at times, unfruitful discussions at the expense of productivity. At times we wished the lead researcher would steer discussions to a greater extent, as meetings often lasted several hours. The lead researcher explained that he felt a need to give us all ample time to discuss the various research topics, in particular when we were up to 10 people sitting around the table. Discussions could ‘float’ for a while to gain new perspectives, but it was important that the lead researcher then focused the discussion and we could agree on decisions. To work effectively as a team, we agreed on the topics for discussion at individual meetings, and also ensured clarity of roles and tasks in the project overall.

### 3.4 Diversity in representation to expand the perspectives of ‘the adolescent voice’

It was important to the project that adolescents' voices were heard as part of the research process, to contribute to research aiming to strengthen the quality of healthcare services for adolescents with mental health problems and conditions. The secondary school contacted, had adolescents with a wide range of different ethnocultural backgrounds, thus allowing a variety of perspectives to be heard. As it turned out, the research group includes adolescents not only of different ethnic and cultural backgrounds, but also both binary genders, different sexual orientations and different life and health experiences. The diversity contributed to a broad range of and richness in experiences and perspectives, which appealed to a diverse audience of adolescents.

However, despite bringing different ‘adolescent voices’ to the project, we are aware that we cannot speak on behalf of all adolescents and we share some core values and opinions, such as our understanding of the importance of academic education. This means we have not yet included representatives from the population of adolescents who are not so interested in education. This was also a reason why we sought other adolescents’ opinions through surveys and at the mental health seminar. Our aim was to gain better insight into diverse experiences and perspectives on mental health services. We think that the research project should continue to strive to hear the voices of those groups of adolescents who have not been heard. This may include for example those who drop out from school and adolescents of different minority groups.

### 3.5 Self‐determination—supporting adolescents' involvement in decision‐making processes

As lead researcher, my views were influenced through over 25 years of experience in private clinical practice outside the public healthcare service (complementary therapies), during which I met many adolescents with mental health problems. They had seen several healthcare practitioners within the primary and secondary healthcare services. Most of them had been brought to me and to other therapists by their parents, in the hope that someone could ‘help to fix their children’. Adolescents were only to a limited extent or not at all involved in the decision‐making process, which was in conflict with my fundamental belief in the individual's right to influence her or his own life. This influenced my view that adolescents' opinions should be well integrated from the first stages of the research project. I was however uncertain to what extent and in which ways adolescents would want and be able to be involved in the project. Their contributions have exceeded my expectations, as they have managed to participate in planning and developing the research, recruiting participants, collecting and analysing data and disseminating results. We engaged in discussions through 80 meetings and over email, and they were involved in various decision‐making processes.

One challenge associated with the principle of self‐determination was the recruitment process. Most adolescents were invited to join the research project during three secondary school meetings. They expressed their interest to the school's management, which selected those who they thought could participate. Hence, adolescents volunteered to participate, but the school served as a gatekeeper for adolescents' participation in the project. This was agreed between the researcher and the school, due to concern that participation might potentially be challenging to adolescents who were currently struggling with their own mental health issues. We, the coresearchers, later discussed the recruitment process and although we were happy that we were selected, we believed that adolescents should in principle have the right to determine whether they have the capacity to participate, following discussions with the researcher.

Another example was the use of adolescents' title. Initially, the term ‘user representatives’ was used, which the first two adolescents who joined the project instantly rejected. They explained that ‘*we do not want to use this term, as it may suggest that we are current users of mental health services, which we are not*’. We jointly agreed that we wanted to limit any confusion about their roles, and to ensure that it would be understood by all parties, as well as ‘the outside world’. This resulted in the decision to use the title ‘adolescent representatives’.

### 3.6 Flexible and systematic project management

There was initial uncertainty as to what extent and in which ways adolescents would be interested in and able to participate in the research project, considering tight schedules with commitments within and outside school. We, the adolescents, had to somehow be given the freedom to be as involved in the project as we could and wanted to; and at the same time familiarize ourselves with each other and the research project. The process included a great deal of flexibility in terms of planning the research, agreeing roles and tasks and practical details, such as agreeing on times and venues for meetings. This flexibility was helpful to us to facilitate inclusion in the project. For example, while planning the mental health seminar, we met later in the day and during weekends to schedule around the school day. The researchers needed to be willing to make this type of ‘sacrifice’ to involve us in their research. However, flexibility also meant that it was more difficult to set short‐ and long‐term goals that could have helped to develop the project faster. Careful thought is needed to balance inclusiveness with effectiveness.

As we have grown older and finalized secondary school, we have moved on and started various university studies in different cities and countries. We have agreed to continue to participate as coresearchers in the project, but our focus in life, the geographical distance and now also limitations set as a result of the COVID‐19 pandemic require greater efforts to organize face‐to‐face meetings and increased use of digital platforms and chat solutions. Greater geographical distance and the limitations imposed through the pandemic have also forced me, the lead researcher, to align my digital profile and competence to those of the coresearchers, for example by using new apps to stay more regularly in communication.

## 4 DISCUSSION

This study adds to the limited research evidence reporting on adolescents' involvement in mental health research.^6^ The small amount of research published in this field stands in contrast to the increasing expectations that researchers should involve members of the public in their research. In some instances, the involvement of patients or the public has even become a requirement, for example, for some funding applications. In our experience, most adolescent mental health research projects only involve youth in limited ways such as asking for their input to interview guides. Such involvement can be helpful but may risk missing out on the full potential benefits of more extensive involvement and it may in some instances also become tokenistic.

So what are helpful ways of ensuring meaningful and effective forms of collaboration with adolescents or youth in mental health research? We propose a 10‐point checklist as a basis for recommendations (Table 2). Its content may be of help for research institutions developing involvement strategies, education providers to develop teaching programmes for researchers and for researchers and adolescents who engage in collaboration. Although our focus is in the field of mental health research, the recommendations might be transferable for other fields of health and social care research. We recognize that a brief checklist carries a potential risk of simplification and misinterpretation of its content. Therefore, we advise researchers and adolescents alike to carefully reflect on the relevance and applicability of the recommendations to their needs, aims and resources.

**Table 2:** Recommendations for involving adolescents in mental health research

1. *Expand researchers' knowledge and competence about adolescent involvement in research*. Examples: Part of master students' and PhD candidates' curriculum, and additional training for their supervisors and other researchers. 2. *Consider the ethical implications of involving adolescents in the research, including to ensure that all parties are aware of everybody's rights and responsibilities*. 3. *Consider the need for involvement and shared decision‐making power at different stages of the research*. Agree on consultation, collaboration or control of the research. 4. *Explore researchers' and adolescents' motives for participating, including their values, to establish a common starting point*. This may also require consideration of the ‘research culture’ within scientific communities, and potentially a need to change the culture. 5. *Provide appropriate training and support for adolescents*. Examples: Seminars providing an introduction to research, mental health research, research design and methods. Use a variety of approaches for communication, for example, digital tools, such as WhatsApp and Messenger. 6. *Researchers' and coresearchers' willingness to contribute and agreement on adolescents' roles and extent of involvement*. Examples: Sharing experiences and perspectives, expanding own knowledge and taking on tasks. This may lead to decisions of whether adolescents participate as representatives or coresearchers. 7. *Establish a good collaborative relationship and reduce power differentials*. Spend time together to build a trusting relationship and reduce feelings of uncertainty. Avoid using research jargon. 8. *Provide sufficient resources, including funding and time to enable collaboration*. Funding for remuneration, compensation of expenses (e.g., for travel), meetings and research activities. 9. *Ensure diversity in adolescent representation suitable for the research project*. Examples: Different cultural backgrounds, genders, sexual orientation, education, life experience and attitudes/approaches towards mental health. 10. *Ensure flexible and effective project management, to ensure that goals are reached and everyone's time and efforts are valued*. Examples: Plan ahead of time, but adapt. Facilitate encouraging meetings. Adapt meeting times and venues to accommodate adolescents’ school, work and other activities/obligations (e.g., meet after working hours, weekends). Food and fun activities during meetings. Conflict management.

Our recommendations require careful consideration if they are to be effectively implemented. For example, assessment of current curricula and teaching needs assessment for master and PhD students might be helpful to expand researchers' knowledge and competence in this field. Furthermore, exploring established researchers' motives may also require consideration of their ‘research culture’. Thompson et al.^17^ found over a decade ago that researchers may be reluctant to involve members of the public in their projects as this might require new ways of thinking and working. A change in research culture would require researchers and adolescents alike to consider their values and motives for carrying out the research. Gradinger et al.^18^ presented normative, substantive and process values that can be associated with public involvement in health and social care research. We found considerable similarities between our reflections and their framework. For example, our theme of commitment and inclusiveness addresses values of openness and honesty suggested by Gradinger et al.^18^ Self‐determination addressed rights values, such as independence and empowerment. Our efforts to reduce power differentials are in line with values of partnership, equality, respect and trust. Flexible and systematic project management addressed values of clarity. Finally, the theme focusing on diversity in representation may address several values, such as representativeness, respect, trust and relevance.

Ethical implications of involving adolescents in the research should also be considered. Some have expressed concern about the impact involvement may have on youths' own mental health.^8^ Although we agree that this may be a concern for some adolescents, we have not experienced any negative impacts. We suggest that adolescents should be allowed to participate to the extent that they feel they can manage; that researchers and adolescents could regularly check if everyone is doing well and appropriate support systems are in place. Another ethical aspect that may need consideration is confidentiality and informed consent. This should also be discussed between researchers and adolescents, and others also propose there could be specific training to address such issues.^5^ Moreover, the consequences of not involving adolescents in mental health research should be considered. Carrying out studies or developing interventions that are not relevant or acceptable to adolescents would be a waste of financial and human resources, and risks further damaging their perception of mental health services.

We have emphasized the importance of establishing a good collaborative relationship, characterized by openness and genuine interest from all participants, and reducing power differentials. This requires a willingness to engage in such a project, and sufficient resources including sufficient time. This may be a challenge in research projects that are carried out over a short period of time. An option is for research organisations to establish panels consisting of adolescents who may be invited to consider multiple research projects. However, the authors of a recently published anthology question whether the involvement of service users contributes to producing research that helps to bring forward the clinical field.^19^ They found that too many resources are used by researchers to establish a relationship where service users feel safe and respected, at the expense of developing new and helpful knowledge. We agree there should be helpful outcomes of the collaboration, but this does not negate the importance of establishing a good relationship between researchers and adolescents. Moreover, consideration of what ‘helpful knowledge’ means is also needed. Academic researchers place significant importance on peer‐reviewed scientific publications, which in time may contribute to developing the services. Although this is an important part of the research, it takes time to reach service end‐users. Organizing a mental health seminar for adolescents, on the other hand, helped deliver information and directly reach young people with healthcare needs.

Diversity in representation should be carefully considered, and efforts should be made to include groups of adolescents whose voice is often not heard. For example, researchers could together with adolescents attempt to develop more innovative recruitment strategies and ‘unconventional’ arenas for recruitment to include those who have dropped out of school and who have not engaged in work life, or those coming from ethnic or sexual minority groups.

Perhaps most importantly, characteristics of individual research projects, such as the aims of projects, the clinical areas and resources such as time, will require researchers and coresearchers (or representatives) to carefully consider each suggested recommendation for involving adolescents in mental health research.

## 5 STRENGTHS AND LIMITATIONS

The main strength of this article is the joint collaboration between the researcher and the coresearchers, with extensive contributions from coresearchers through all phases of the research. Hence, the results provide a report by, rather than about, coresearchers. This was more in line with the core values of the group and the agreed principles of involving adolescents in all phases of the research. The addition to this study of other researchers and clinicians who were involved in the overall research project could have influenced the overall results. However, the main focus was to emphasize the voices of coresearchers and the researcher who worked most closely with them. The use of an autoethnographic approach meant that coresearchers were reflecting without the ‘interference’ of a researcher and the results were also interpreted by them, rather than by researchers. The fact that the autoethnographic approach was relatively time‐consuming may be considered a disadvantage, but this could also be seen as an advantage as the process contributed to further strengthening the collaborative relationship within the group.

The fact that adolescents did not have an academic research background could be considered a limitation. They did however receive some training from the project lead who is an experienced researcher, and who also provided guidance for the autoethnographic approach and in writing this article. Most coresearchers speak English well, which also helped to facilitate the writing process.

As previously stated, a limitation of this study is that we have not involved adolescents who are rarely included in research projects, such as those who drop out of school and work life. The coresearchers do however have a variety of different ethnic backgrounds, of both genders and with different life and healthcare experiences.

## 6 CONCLUSION

This article adds to the limited research literature reporting on adolescents' active involvement in mental health research. Six themes developed through self‐reflective and group processes describe a collaborative relationship between researchers and adolescents, including personal and group characteristics, such as commitment, inclusiveness, support, flexibility and sharing decision‐making power. Based on our experiences, we provide a 10‐point checklist with recommendations for involving adolescents in mental health research, as a starting point for research institutions, education providers and last but not least—for researchers and adolescents. We believe the implementation of these recommendations could contribute to a change in the ‘research culture’ and thereby strengthen the involvement of adolescents in research, further observing their basic rights as set out by the United Nations and Council of Europe.

## CONFLICT OF INTERESTS

The author declares that there is no conflict of interest that could be perceived as prejudicing the impartiality of the research reported.

## AUTHOR CONTRIBUTIONS

Petter Viksveen, Julia R. Game and Nicole E. Cardenas initiated the development of the research and developed the initial project plan. The remaining authors contributed to planning the research. All authors contributed to the data collection process and the data analysis. Petter Viksveen led the manuscript development and all coauthors contributed. The final manuscript was approved by all authors.

## Supporting information

Supplementary information.

Supplementary information.

## Data Availability

Data are available on request due to privacy/ethical restrictions. The data that support the findings of this study are available on request from the corresponding author. The data are not publicly available due to privacy or ethical restrictions.
