# Supplementary material for: Involvement of adolescent representatives and coresearchers in mental health research: Experiences from a research project
Source: Health Expect. 2021 Nov 10;25(1):322–32. doi: 10.1111/hex.13383 (PMC8849237; doi:10.1111/hex.13383)
Supplement: Supplementary file 1 — Supplementary information. [file HEX-25-322-s002.docx]

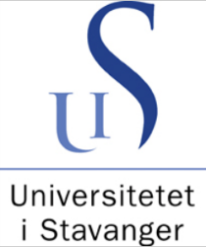


Agreement for cooperation for a research project initiated and run by the University of Stavanger: The InvolveMENT project

**Background**

This agreement has been developed through a consensus process with the participation of all signatories in order to set the terms for cooperation between researchers at the University of Stavanger (hereinafter ‘the researchers’) and student at [name of school]. The researchers are carrying out research in the field of adolescents’ mental health. [name of school] is in this agreement represented by the [program lead].

As part of the research, the student has been invited to participate as a representative/co-researcher [delete as appropriate throughout the agreement] as part of the research team. The project will also consist of healthcare practitioners as researchers and a number of representatives from e.g. user organizations. The involvement of various stakeholder groups with advisory roles is crucial throughout all phases of the research project.

The long-term plan for the research project includes: setting up a cohort (group) of adolescents for assessing their mental health (irrespective of whether they have healthcare challenges or not) and use of healthcare services; assessment of user involvement for adolescents’ mental health; and development and testing of interventions to support adolescents’ mental health and their use of healthcare services. The plan may change, subject to input by the various stakeholder groups, by discretion of the researchers and representatives/co-researchers, or due to any external circumstances (e.g. financial support).

**Roles and responsibilities**

The University carries the rights and responsibilities for the research project, including any financial and academic aspects directly related to the research. The researchers may thereby, at their own discretion, make any final decisions concerning the research and have the ownership of the academic material produced as part of the research project, but will consult with representatives/co-researchers and other stakeholder groups included in the research project. The researchers will provide information to contribute to representatives’/co-researchers’ understanding of the research project. The researchers are not in a position to provide representatives/co-researchers with psychological support, but can make recommendations for where they may obtain such support by their own request or if the researchers have any major concerns for their wellbeing.

The student has been invited to and has accepted to take on the role as representative/co-researcher as part of the team that is planning and overseeing the research project. This may include any of a number of different phases of the research project, ranging from the initial stages of developing the research project, the research priorities and strategies, the applications for funding and ethics approval, monitoring and assessing the research project, and disseminating the results of the research. Representatives/co-researchers will be asked about and may contribute with their ideas, views, opinions and proposals for the research project. Representatives/co-researchers may be also be engaged in other research-related activities, for example development of and running surveys, data analysis, developing guidelines and recommendations, writing articles for submission to popular and scientific media, participating and presenting at or running workshops, seminars or conferences focusing on topics relevant to adolescents’ mental health.

Representatives/co-researchers are free to leave the project at their own discretion and without giving a reason. They are responsible for and have independent ownership of any assignments that they may decide to carry out as part of their studies at [name of school]. Any agreements for such assignments will be made between the student and the school. However, in the event that such an assignment includes information on the basis of the University research project, the researchers must be consulted and give their permission for publication of results. The school holds the responsibility for any follow-up of the student’s academic development, but the researchers may provide students with information to further their knowledge in the field of research (e.g. to gain an understanding of the process of development and running of a research project and to obtain a basic understanding of qualitative and quantitative research methods).

Confidential information obtained through the research project may not be disclosed to any other parties.

**Financial issues and certificates**

The school and the representatives/co-researchers are not expected to contribute financially to the research project. The University of Stavanger will cover travel expenses (standard local public transport fares, with receipts) to attend any scheduled meetings. In addition, a nominal hourly fee is paid for each scheduled meeting the student attends, provided this is not in conflict with the school’s regulations. The fee is agreed on an annual basis, currently in the amount of NOK [amount] per hour for representatives/co-researchers at the age of [insert age]. Fees are paid and expenses covered for meetings directly relating to the research project and need to be agreed with the researcher(s), but not for any additional visits (e.g. if the student is invited to attend lectures or working group sessions). Fees may be revised on an annual basis.

The University’s researcher will by the end of the cooperation, or earlier if needed, provide a certificate to describe the student’s contribution to the research project.

-------------------------------------- Date:

[project lead]

On behalf of the University of Stavanger

-------------------------------------- Date:

[student]

[name of school]

[name of school] is familiar with and has no objections to this agreement.

-------------------------------------- Date:

[name of responsible person]

[name of school]

Each signatory holds a copy of this agreement. The original copy is kept by the University of Stavanger, Faculty of Health Sciences, Kjell Arholms Hus, Kjell Arholms gate 39, 4021 Stavanger.
