# Supplementary material for: Involvement of adolescent representatives and coresearchers in mental health research: Experiences from a research project
Source: Health Expect. 2021 Nov 10;25(1):322–32. doi: 10.1111/hex.13383 (PMC8849237; doi:10.1111/hex.13383)
Supplement: Supplementary file 2 — Supplementary information. [file HEX-25-322-s001.docx]

**Appendix B. Questions for the self-reflective processes in the research project**

All questions are phrased for adolescent representatives and co-researchers. All questions relevant to the lead researcher were used for the researcher’s own self-reflective processes. The key questions were answered by all. The additional questions addressing various aspects were only answered by each individual co-researcher and the researcher if they were considered relevant. Responses could be in a narrative form, i.e. to give each person an opportunity to “tell their story”.

**Key questions about your involvement in the research project**

What were your first thoughts when you heard about the project?

What did you first think about becoming an adolescent representative?

What was best about being involved in the project?

What were the challenges of being involved in the project?

**Additional questions addressing various aspects of the research project**

**Preparation for involvement in the project**

What do you think the greatest weakness of preparing you for the project were?

What should the researchers have done to better support you in your roles as representative and co-researcher?

What should the researchers have done differently or more of to ensure you felt sufficiently confident to deal with the various research topics and methods?

What did the researchers do well?

**Communication and collaboration**

How was the communication and cooperation with the researcher and the research centre?

What was particularly good about it? What was difficult or challenging?

How could collaboration between researchers and the school/teachers be improved?

In which ways did you communicate to others and who did you communicate with, as part of doing this research? This may be e.g. just chatting with family, friends, peer students, teachers or others.

Did any of your peer students or others contact you out of curiosity or with questions about your participation in the project or the project itself?

**Roles**

How did you feel the different titles “adolescent representative” and “adolescent co-researcher” affected your understanding of your roles and your involvement in the project?

What were the challenges of balancing being involved in the project and carrying out your studies?

How did participating in the project contribute to your own studies at your school?

What was the school’s role and how did this work? What ran smoothly and what was difficult?

**Values**

What are the motives and values of co-researchers and researchers for joining this project?

Were there any agreed values or principles that guided the work together?

What are important values required for researchers, representatives/co-researchers, and the collaboration?

What do you want in a team leader?

What do you want in participants/co-researchers?

**Knowledge, competence and career development**

How did this research project contribute to your knowledge and competence?

How did participating in the project contribute to your own studies?

How did you use the knowledge gained?

What did you know about mental health prior to joining the project?

What did you know about research prior to joining the project?

What did you learn during the project?

How did you expand your understanding (teaching by researcher, workshop, other)?

How has your understanding/views/opinions of mental health changed throughout the cooperation?

How has your understanding/views/opinions of research changed throughout the cooperation?

Did participating in this project contribute to your choices for your future career? If so, how?

**Recommendations for future involvement**

What are your recommendations to teenagers who consider being involved in such a project?

What are your recommendations to researchers who consider involving adolescents?

How do we effectively involve teenagers as representatives or co-researchers in a mental health research project?

What should be thought about beforehand? What should be done during?

What should the requirements be for those who are invited as representatives and co-researchers?

What would be reasons not to involve teenagers in such a role?

How should we agree on how teenagers are involved in the project?

How would you like to participate in the project in the future (after you leave your school)?

What would you like to do more of? What would you prefer to avoid?

**Use of titles**

How did you come to the suggestion to change your title from representative to co-researcher?

How do you feel the different titles have affected your perception of your roles and your involvement in the project?

**Roles and communication activities**

What was your role in the planning of the University’s large research projects?

How did you experience the processes of developing the applications for funding?

What do you think your role could or should be in carrying out the research?

How did you come to think about running a survey among peer students?

What did you originally think you would get out of this? What did you get out of it?

How can this knowledge be used? Who can it be presented for and in which ways?

What was your role in the planning of the systematic review?

In which way(s) were you included in writing the protocol article?

What is your role in carrying out the review? What are the greatest challenges in doing this?

How do you think you can contribute to expand the researchers’ perspectives?

What was your experience with the meeting with the Minister of Health and the Minister og Children and Equality? Did it influence you in any way?

**Focus of research**

What did you think about the focus of the project?

What should we focus more or less on in the research?

If we could get unlimited funding, what should be the main areas of focus in the research?
